# Prevalence and subtypes of Influenza A Viruses in Wild Waterfowl in Norway 2006-2007

**DOI:** 10.1186/1751-0147-52-28

**Published:** 2010-04-28

**Authors:** Anna Germundsson, Knut I Madslien, Monika Jankowska Hjortaas, Kjell Handeland, Christine Monceyron Jonassen

**Affiliations:** 1 Department of Animal Health, National Veterinary Institute, P.O Box 750 Sentrum. N-0106 Oslo, Norway; 2 Center for Laboratory Medicine, Akershus University Hospital, N-1478 Lørenskog, Norway

## Abstract

The prevalence of influenza A virus infection, and the distribution of different subtypes of the virus, were studied in 1529 ducks and 1213 gulls shot during ordinary hunting from August to December in two consecutive years, 2006 and 2007, in Norway. The study was based on molecular screening of cloacal and tracheal swabs, using a pan-influenza A RT-PCR. Samples found to be positive for influenza A virus were screened for the H5 subtype, using a H5 specific RT-PCR, and, if negative, further subtyped by a RT-PCR for the 3'-part of the hemagglutinin (HA) gene, encompassing almost the entire HA2, and the full-length of the neuraminidase (NA) gene, followed by sequencing and characterization. The highest prevalence (12.8%) of infection was found in dabbling ducks (Eurasian Wigeon, Common Teal and Mallard). Diving ducks (Common Goldeneye, Common Merganser, Red-breasted Merganser, Common Scoter, Common Eider and Tufted Duck) showed a lower prevalence (4.1%). In gulls (Common Gull, Herring Gull, Black-headed Gull, Lesser Black-headed Gull, Great Black-backed Gull and Kittiwake) the prevalence of influenza A virus was 6.1%. The infection prevalence peaked during October for ducks, and October/November for gulls. From the 16 hemagglutinin subtypes known to infect wild birds, 13 were detected in this study. Low pathogenic H5 was found in 17 dabbling ducks and one gull.

## Findings

Birds of wetlands and aquatic environments constitute the major natural reservoir of influenza A viruses of all hemagglutinin (HA) and neuraminidase (NA) subtypes (H1-H16 and N1-N9) [1,2]. In particular, birds belonging to *Anseriformes *(ducks, geese and swans) and *Charadriiformes *(gulls, terns and waders) have been reported to be efficient hosts. The birds do not usually develop clinical disease, but they shed a large number of virus particles in their faeces, which may cause serious disease outbreaks when introduced into poultry flocks. The prevalence of avian influenza A viruses in their natural hosts depends on geographical location, season, year and host species. For instance, in Sweden the prevalence of influenza A viruses in Mallards were 3-fold higher as compared to the Netherlands during the same time of the year [3]. Following the outbreak of highly-pathogenic avian influenza (HPAI) H5 at Qinghai Lake in China in 2005, where 10 000 wilds geese and ducks died, there has been an increased focus on wild birds as carriers of the HPAI H5 contributing to geographical spread of the virus, and as source of infection for poultry [4-6]. In Norway, an active surveillance program on influenza A viruses in wild waterfowl was started in 2005 [7]. In this study, we present the results of this program during the subsequent years 2006 and 2007.

**Figure 1:**
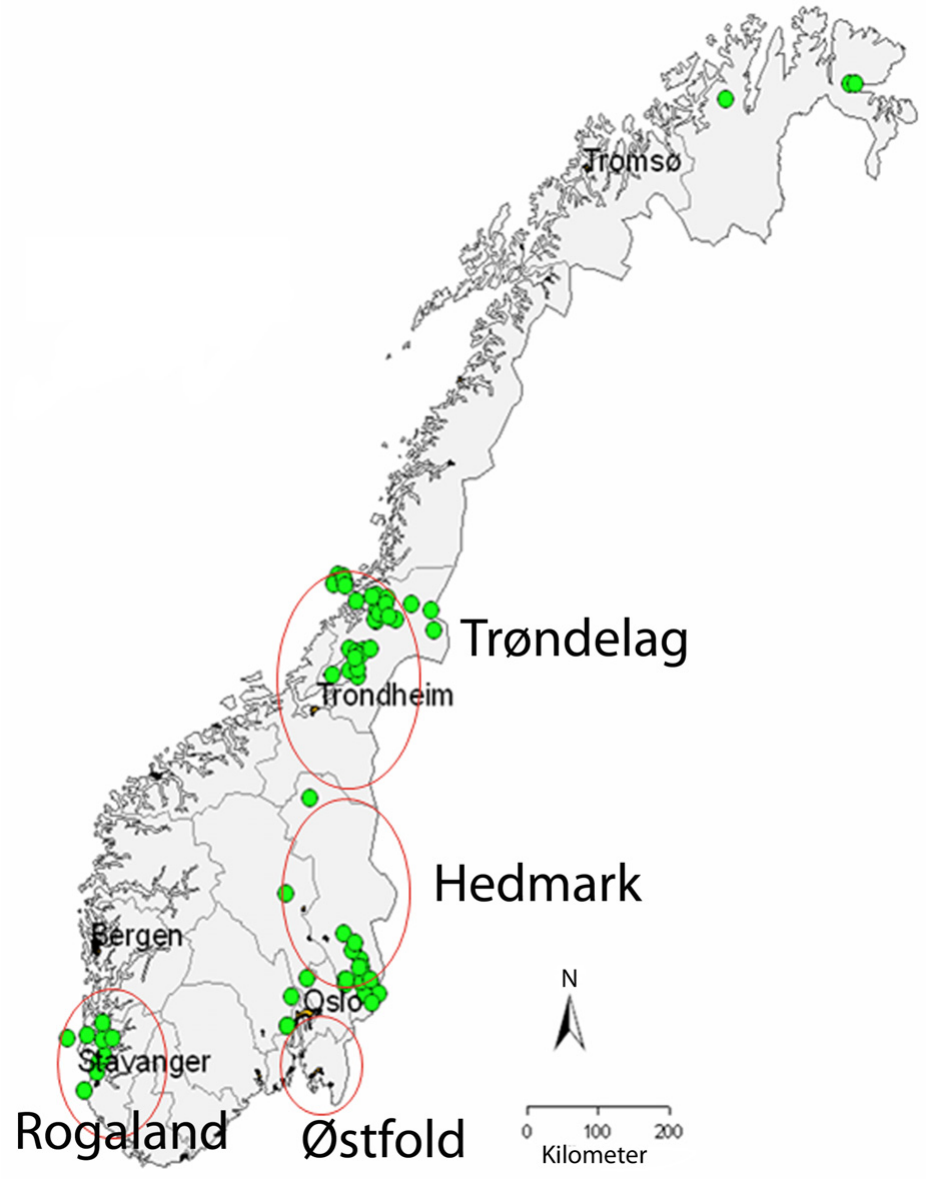
**Geographical location of sampling regions (counties) for wild waterfowl examined for influenza A virus in Norway in 2006 and 2007**. The red rings illustrate locations where birds were sampled. The green spots show important stop-over locations for migrating ducks.

Cloacal and tracheal swabs were collected from a total of 2742 birds. The sampling included 1480 samples from three species of dabbling ducks, 49 samples from six species of diving ducks and 1213 samples from six gull species (Table 1). The samples were collected from birds shot during the licenced hunting season from August to December in 2006 and 2007, in four different counties in Norway known to have high densities of poultry and being important stop-over locations for migrating ducks (Figure 1). From each bird, cloacal and tracheal swabs were collected, pooled by placing the two swabs in the same virus transport medium and sent to the laboratory by postal mail. At arrival in the laboratory, 200 μl of the medium were used for RNA extraction and the rest was stored at -80°C. RNA was extracted using the automatic extraction instrument NucliSens^® ^easyMag™ (bioMérieux bv, Boxtel, The Netherlands) according to the manufacturer's instruction, and eluted in 55 μl. Detection of influenza A virus was performed using primers and probe targeting part of the 5'-end of the Matrix gene [8]. Amplification was performed on a Stratagene Mx3500P (LaJolla, CA, USA) using the Qiagen One-Step RT-PCR kit (Qiagen, West Sussex, UK), with 0.4 μM of each primer, 0.3 μM of probe, and a MgCl_2 _concentration of 1.25 mM. The RT step was run for 30 min at 50°C, followed by 15 min at 95°C. A three-step PCR cycling protocol was used using the following conditions: 45 cycles of 94°C for 15 s, 55°C for 30 s and 72°C for 15 s. Samples with a ct-value of 38 or below were considered to be positive for influenza A virus. Influenza A positive samples were further tested for H5 subtype [9]. Samples found to be positive for influenza A virus, but negative for subtype H5, were subtyped by performing RT-PCRs and sequencing for the HA2 and full-length NA genes [10,11]. The nucleotide sequences obtained in this study were deposited in the EMBL database (EMBL: FM179753-FM179764, EMBL:FN773066-FN773082). A few samples were selected for virus isolation in embryonated chicken eggs (data not shown).

**Table 1:** Overview of wild waterfowl sampled for influenza A virus examination in Norway 2006 and 2007

	Species		No. of birds analysed 2006	No. of positive birds (%) 2006	No. of birds analysed 2007	No. of positive birds (%) 2007
Dabbling ducks	*Anas penelope*	Eurasian Wigeon	137	4(2.9)	119	12(10.1)
	*Anas crecca*	Common Teal	100	6(6.0)	238	38(15.9)
	*Anas platyrhynchos*	Mallard	359	50(13.9)	527	79(14.3)
Diving ducks	*Bucephala clangula*	Common Goldeneye	15	0	4	0
	*Mergus merganser*	Common Merganser	7	0	0	0
	*Mergus serrator*	Red-breasted Merganser	6	0	1	0
	*Melanitta nigra*	Common Scoter	2	0	1	0
	*Somateria mollissima*	Common Eider	0	0	9	2(22.2)
	*Aythya fuligula*	Tufted Duck	0	0	4	0
Gulls	*Larus canus*	Common Gull	173	6(3.5)	211	19(9.0)
	*Larus argentatus*	Herring Gull	363	10(2.8)	328	30(9.1)
	*Larus ridibundus*	Black-headed Gull	19	4(21.1)	11	1(9.1)
	*Larus fuscus*	Lesser Black-headed Gull	8	0	0	0
	*Larus marinus*	Great Black-headed Gull	34	2(5.8)	64	2(3.1)
	*Rissa tridactyla*	Kittiwake	2	0	0	0

**TOTAL**	**Dabbling ducks**		**596**	**60(10.1)**	**884**	**129(14.6)**
**TOTAL**	**Diving ducks**		**30**	**0**	**19**	**2(10.5)**
**TOTAL**	**Gulls**		**599**	**22(3.7)**	**614**	**52(8.5)**

The number of birds examined and found virus positive (%) are given for each species.

The prevalence of influenza A virus in wild birds in Norway in 2006 and 2007 are presented in Table 1. High prevalence of infection was found in dabbling ducks (189/1480, 12.8%), whereas lower prevalence were seen in diving ducks (2/49, 4.1%) and gulls (74/1213, 6.1%). The finding of a higher prevalence in dabbling compared to diving ducks is consistent with results found in other studies [2,3,12]. Virus in faeces from infected birds is excreted into the surface water and may more efficiently be transmitted to dabbling ducks feeding there, as compared to diving ducks feeding at deeper water levels. It has been shown that influenza viruses can remain infectious in the surface water for several days [13,14].

The prevalence of influenza A virus in wild birds varied between the two years of study. In Mallards the prevalence was similar, 13.9% (50/359) in 2006 and 14.9% (79/527) in 2007. In Common Teal the prevalence altered from 6% (6/100) in 2006 to 15.9% (38/238) in 2007, and in gulls from 3.7% (22/596) in 2006 to 8.5% (52/614) in 2007 (Table 2). In 2005, the prevalence in Mallards and Common Teal were of 20.4% (58/284) and 30.9% (13/42) respectively [7]. A possible explanation for the lowered observed prevalence in 2006 could be due to climatic variations. The summer of 2006 was exceptionally warm, and especially the water temperature in lakes and sea was elevated. It has been shown that the survival of influenza A virus in water decreases for water temperature above 17 degrees, that only rarely are achieved in lakes in Norway [13]. In both sampling years, the highest prevalence for ducks was seen in October, whereas in gulls the peak prevalence varied between October (2006) and November (2007) (Table 2). The high prevalence seen in ducks in October may be a result of the close contact, and possibility of virus transmission between individuals, following the dense aggregation of these birds along their migratory route towards wintering areas.

**Table 2:** Prevalence of influenza A virus, separated by month, in wild waterfowl in Norway 2006-2007.

Year Month	2006 Ducks No. of positive/ No. of tested	2006 Gulls No. of positive/ No. of tested	2007 Ducks No. of positive/ No. of tested	2007 Gulls No. of positive/ No. of tested
August	2/102	1/47	14/186	7/123
	1.9%	2.1%	7.5%	5.7%
September	10/100	5/92	30/250	24/270
	10%	5.4%	12%	8.9%
October	23/137	5/68	43/204	7/87
	16.8%	7.4%	21.1%	8.0%
November	23/201	2/101	35/190	13/100
	11.4%	2.0%	18.4%	13%
December	2/86	9/291	9/73	1/34
	2.3%	3.1%	12.3%	2.9%

**TOTAL**	**60/626**	**22/599**	**131/903**	**52/614**
	**9.6%**	**3.7%**	**14.5%**	**8.5%**

The table gives the number of positive samples, the total number of samples tested as well as the prevalence in percent.

**Figure 2:**
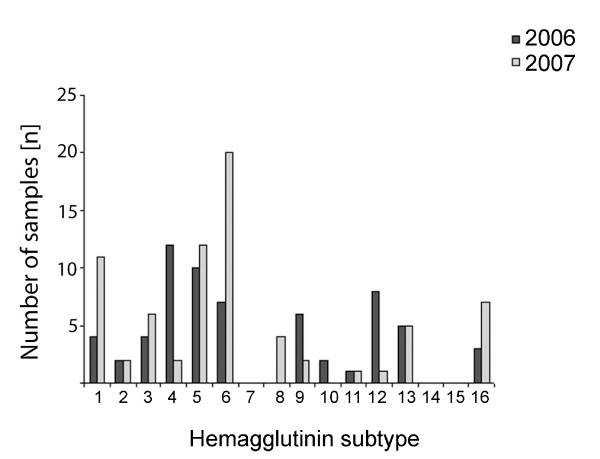
**Comparison of different influenza A virus HA subtypes found in wild Norwegian ducks and gulls sampled in 2006 and 2007**. Note that there were no detection of H7, H14 and H15. Otherwise, all HA-subtypes were represented. H13 and H16 have until now only been detected in gulls.

From a total of 263 birds testing positive for influenza A virus, the HA subtype was successfully determined in 127 samples from ducks and 39 samples from gulls (Figure 2). The subtype H5 was found in 22 birds, and further sequencing of the cleavage site of the HA gene identified all of them as low-pathogenic strains (LPAI). Seventeen of these samples were detected in Mallards, one in Eurasian Wigeons, three in Common Teals, and one in Herring Gulls. A great number of subtypes were detected in ducks; H1-H6 and H8-H12 were detected in Mallards, H1, H3-H6, H8, H9 and H12 in Common Teals, and H1, H5, H6 and H9 in Eurasian Wigeons. The most frequently detected subtypes in ducks in 2006 were H4 and H12, whereas subtypes H1 and H6 were most prevalent in 2007. The H6 subtype was the most common subtype found in ducks in this country in 2005 [7]. The most frequently occurring subtypes found in gulls in the present study were H13 and H16, although H1 and H4-H6 were also randomly found. H13 and H16 have only been found to infect gulls. In Common Gulls subtypes H6, H13 and H16 were detected, whereas subtypes H1, H5, H6, H13 and H16 were found in Herring Gulls, H4 and H13 in Black-headed Gulls, and H4 in Great Black-backed Gulls.

The NA subtype was determined in 78 of the 263 birds that tested positive for influenza A virus. The NA subtypes found in Norwegian wild birds were N1 (5 samples), N2 (29 samples), N3 (6 samples), N5 (2 samples), N6 (14 samples) and N8 (12 samples). All samples were screened and all positive samples were sequenced directly from primary swab material, without prior virus isolation. Such a strategy might result in higher number of positive samples in screening surveys as compared to strategies where virus isolation is performed prior to RT-PCR screening, as it is difficult to isolate virus from samples with low virus titer. However, sequencing of samples without prior virus isolation on samples with low titer is difficult when amplifying large fragments as using generic primers from HA and NA as attempted in this study. Thereby the proportion of subtypes determined in this study is relatively low.

In this study we report a higher prevalence of influenza A virus in wild birds than has been reporter from other countries in Europe [3]. Similar prevalence of infected wild birds has been observed in Sweden and North America [3,15]. This might suggest that the ecological system with breeding areas and temperatures in these countries is favourable for replication of influenza A virus in wild birds and transmission of influenza A virus among the wild birds.

## Competing interests

The authors declare that they have no competing interests.

## Authors' contributions

AG and MJH carried out the real-time RT-PCR, RT-PCR, sequencing analysis, and interpretation of data. KIM was responsible for the logistics and collection of samples. CMJ and KH participated in the design of the study. AG and KH drafted the manuscript. All authors read and approved the final manuscript.
